# Proteomic Profiling of Sugar Beet (*Beta vulgaris*) Leaves during Rhizomania Compatible Interactions

**DOI:** 10.3390/proteomes2020208

**Published:** 2014-04-09

**Authors:** Kimberly M. Webb, Carolyn J. Broccardo, Jessica E. Prenni, William M. Wintermantel

**Affiliations:** 1 USDA-ARS-SBRU, Crops Research Laboratory, 1701 Centre Ave., Fort Collins, CO 80526, USA; 2 Proteomics and Metabolomics Facility, Colorado State University, C130 Microbiology, 2021 Campus Delivery, Fort Collins, CO 80523, USA; E-Mails: Carolyn.Broccardo@colostate.edu (C.J.B.); Jessica.Prenni@colostate.edu (J.E.P.); 3 USDA-ARS-CIPRU, 1636 E. Alisal St., Salinas, CA 93905, USA; E-Mail: Bill.Wintermantel@ars.usda.gov

**Keywords:** *Beet necrotic yellow vein virus* (BNYVV), shot-gun proteomics, sugarbeet

## Abstract

Rhizomania, caused by *Beet necrotic yellow vein virus* (BNYVV), severely impacts sugar beet (*Beta vulgaris*) production throughout the world, and is widely prevalent in most production regions. Initial efforts to characterize proteome changes focused primarily on identifying putative host factors that elicit resistant interactions with BNYVV, but as resistance breaking strains become more prevalent, effective disease control strategies will require the application of novel methods based on better understanding of disease susceptibility and symptom development. Herein, proteomic profiling was conducted on susceptible sugar beet, infected with two strains of BNYVV, to clarify the types of proteins prevalent during compatible virus-host plant interactions. Total protein was extracted from sugar beet leaf tissue infected with BNYVV, quantified, and analyzed by mass spectrometry. A total of 203 proteins were confidently identified, with a predominance of proteins associated with photosynthesis and energy, metabolism, and response to stimulus. Many proteins identified in this study are typically associated with systemic acquired resistance and general plant defense responses. These results expand on relatively limited proteomic data available for sugar beet and provide the ground work for additional studies focused on understanding the interaction of BNYVV with sugar beet.

## 1. Introduction

Rhizomania, a disease that reduces root quality and yield in sugar beet (*Beta vulgaris*) is one of the most widely prevalent and economically important diseases affecting sugar beet production throughout the world [1,2,3]. Rhizomania is caused by *Beet necrotic yellow vein virus* (BNYVV) [4,5] and is transmitted via the plasmodiophorid *Polymyxa betae* [6]. Fields can remain infested with BNYVV indefinitely as *P. betae* cystosori can remain dormant for up to 25 years [4,7]. Therefore, typical remediation approaches such as rotation to non-host crops or lengthening rotations are ineffective at reducing disease incidence, leaving host-plant resistance as the only economically viable means of control [3].

A number of single dominant resistance genes (known as *Rz* genes) have been identified for control of BNYVV beginning with the discovery and introgression of the *Rz1* resistance gene [8,9], which became widely planted throughout all areas where rhizomania threatens sugar beet production. Over the past two decades additional resistance genes have also been identified [10,11]. The different sources of resistance genes appear to have different underlying mechanisms, which are largely undetermined [1,12,13] and several uncharacterized minor genes may contribute to enhanced resistance associated with the primary *Rz* genes [1]. Although the widely used *Rz1* gene prevents symptom development, the virus can still replicate at a low level in resistant plants. This has resulted in emergence of BNYVV variants that can accumulate enough in the presence of the resistance gene and overcome *Rz1* resistance in the field.

Following the introduction of resistant sugar beet varieties containing the single dominant *Rz1* gene, new pathotypes that overcome resistance emerged after only a few cropping seasons [5]. Three major BNYVV types have been reported world-wide; A-type, B-type, and P-type [14,15,16], with the A-type distributed throughout most sugar beet growing regions of the world [17]. The B-type is predominantly restricted to France and England [16] although limited identifications of the B-type have occurred in Sweden, China, Japan and Iran (summarized in [3]). The A and B types of BNYVV each contain 4 viral RNAs, with RNAs 1 and 2 encoding proteins involved in viral replication, and encapsidation and cell-to-cell movement, respectively. RNA3 encodes a protein that affects pathogenicity and determines the ability of the virus to overcome the most common source of genetic resistance to the virus. RNA4 encodes a single protein involved in transmission by *P. betae* (reviewed in [18]). The P-type is closely related to the A-type, but contains a fifth RNA [17,19]. Isolates containing a fifth RNA usually have increased symptom severity [20]. More importantly, the P-type is able to overcome the *Rz1* resistance gene, which is used to control BNYVV throughout the world. To date the P-type has been restricted to portions of France, Kazakhstan, England, and Iran [15,21,22,23]. All American isolates identified to date have been A-type, and until a decade ago these were effectively controlled by varieties carrying the *Rz1* gene [8]. In 2002 and 2003, resistant sugar beet in the Imperial Valley of California carrying the *Rz1* resistance gene developed severe rhizomania symptoms due to emergence of a mutant variant of the A-type, now known as the Imperial Strain (BNYVV-IV), which contains two amino acid mutations in the P25 protein encoded by RNA3 that allow the virus to overcome *Rz1* sources of resistance [5]. Subsequent studies have identified similar resistance-breaking variants throughout American sugar beet production regions, although most are limited in distribution [24]. Little is known of the interactions between sugar beet and BNYVV that influence epidemiology, and until the various mechanisms of resistance and infection are understood more fully, alternative disease control methods and additional sources of resistance will be required to control this pathogen.

There have been some proteomic analyses in sugar beet that have focused on abiotic agronomic stress conditions such as drought [25], salt stress [26,27], and nutritional deficiencies [28], and some examined tissue specific differences in protein expression [29,30]. Few published accounts focused primarily on characterizing the interaction of sugar beet with plant pathogens [31,32]. Proteomic approaches to characterize the interaction of BNYVV with sugar beet have been relatively limited. For example, using a subtractive proteomic approach, Larson *et al.* [32] previously identified 50 putative sugar beet proteins that were either up or down regulated in response to infection with a single BNYVV type. These 50 proteins were uniquely expressed in infected plants of a single susceptible genotype at six weeks after planting compared with uninoculated controls. While Larson *et al.* [32] was able to report on proteins that were differentially expressed with a single BNYVV type, due to the lack of a fully sequenced and annotated sugar beet genome and limitations in the ability to detect low abundance proteins in that study, further analysis of the sugar beet proteome was warranted. The goal of this work is to develop methods for exploratory proteomic profiling of susceptible sugar beet leaves. This approach allows for the unbiased evaluation of the detectable proteins in a sample, and lays the groundwork for future quantitative studies between defined treatments. Such an approach provides data for hypothesis generation and serves to better define the hereto poorly annotated sugar beet proteome. We examined the protein expression during compatible (susceptible) interactions with A-type as well as during infection by the Imperial Strain of BNYVV, which overcomes the *Rz1* resistance gene, the most widely used source of resistance to BNYVV in commercial sugar beet germplasm. This was done in order to identify proteins that are expressed during disease development, in the hopes of gaining an understanding of the proteins that may be involved in the underlying plant defense response.

## 2. Experimental

### 2.1. Plant Propagation and Inoculation

A proprietary sugar beet variety, R30_rz1, which is susceptible to all forms of BNYVV, was provided by KWS (Einbeck, Germany). To enable detection of a broad range of proteins associated with compatible interactions, two sources of BNYVV were used; standard BNYVV A-type, originally collected from Spence Field at the USDA-ARS in Salinas, CA (BNYVV-A), and a *Rz1* resistance-breaking mutant variant of the A-type originating from the field, Rockwood 158, Imperial County, CA (BNYVV-IV) [5]. Previous studies indicate that these isolates are virtually identical, differing only by two amino acids in the P25 protein, which allow BNYVV-IV to overcome resistance encoded by the *Rz1* gene [2]. Both isolates are well-characterized and represent the broadest known range of variability expected for North American BNYVV isolates in sugar beet with respect to performance against the widely planted *Rz1* source of resistance to BNYVV. Soil containing either BNYVV-A or BNYVV-IV was mixed with equal parts sterile builders sand, placed in new Styrofoam cups, and 100 R30_rz1 sugar beet seeds were planted per cup, with five cups per treatment. Seedlings were grown as described in Larson *et al.* [32] in a Conviron PGC15 Growth Chamber (Winnipeg, MB, Canada) at 24 °C with 16 h days and approximately 220 µM m^−1^s^−2^ light. At 3 weeks post-germination plants were harvested and washed to remove soil. Seedlings from all five cups for each treatment were pooled, and foliar and root portions of the plant were separated at the crown. Pooled root samples from each treatment were tested by ELISA to confirm BNYVV infection [33]. Only samples with ELISA absorbance readings of at least 2 times the absorbance of healthy controls were subjected to proteomic analysis. Pooled leaf tissue from each treatment was ground in liquid nitrogen and lyophilized. The entire experiment was biologically replicated three times.

### 2.2. Protein Digestion and Mass Spectrometry

Total protein was extracted from 100 mg (dry weight) of lyophilized leaf tissue for each treatment from each biological replicate, using the Plant Total Protein Extraction Kit (Sigma, St. Louis, MO, USA) according to manufacturer’s recommendations. Following extraction, suspended protein was further cleaned for quantification using the ReadyPrep 2-D Cleanup Kit (Bio-Rad, Hercules, CA, USA) in aliquots of 100 µL following manufacturer’s recommendations. Protease inhibitors were added to liquid protein extracts (Pierce, Rockford, IL, USA), and samples were stored at −80 °C prior to analysis. Protein concentrations were determined via Bradford Assay [34] (Thermo Scientific, Rockford, IL, USA) and 30 µg of each sample underwent in-solution proteolytic digestion as previously described [35]. Briefly, samples were solubilized in 8 M urea, 0.2% Protease Max (Promega, Madison, WI, USA), then reduced with dithiothreitol, alkylated with iodoacetamide, and digested with 1% Protease Max and trypsin at 37 °C for 3 h. Samples were dried in a Speed Vac^®^ vacuum centrifuge, desalted using Pierce PepClean C18 spin columns (Pierce, Rockford, IL, USA), dried and resuspended in 30 µL 3% ACN, 0.1% formic acid. All solvents, water, and acid were LC-MS/MS grade from Sigma (St. Louis, MO, USA). One µL of each sample (from the three biological replicates from each soil type) was analyzed in duplicate injections via LC-MS/MS. Peptides were purified and concentrated using an on-line enrichment column (Thermo Scientific 5 µm, 100 µm ID × 2 cm C18 columns). Subsequent chromatographic separation was performed on a reverse phase nanospray column (Thermo Scientific EASYnano-LC, 3 µm, 75 µm ID × 100 mm C18 column) using a 90 min linear gradient from 10%–30% buffer B (100% ACN, 0.1% formic acid) at a flow rate of 400 nL/min. Peptides were eluted directly into the mass spectrometer (Thermo Scientific Orbitrap Velos Pro) and spectra collected over a *m*/*z* range of 400–2,000 Da using a dynamic exclusion limit of 2 MS/MS spectra of a given peptide mass for 30 s (exclusion duration of 90 s). High resolution MS level scans were collected in the FT (resolution of 60,000), and MS/MS spectra were collected in the ion trap (IT). Compound lists of the resulting spectra were generated using Xcalibur 2.2 software (Thermo Scientific, Rockford, IL, USA) with an S/N threshold of 1.5 and 1 scan/group.

### 2.3. Protein Identification and Data Analysis

MS/MS spectra were searched against a Uniprot *Amaranthaceae* database [36] concatenated to a reverse database and the Uniprot *Mus musculus* (mouse) database [37] using the Mascot database search engine (Matrix Science, version 2.3.2) and SEQUEST (version v.27, rev. 11, Sorcerer, Sage-N Research). Due to the limited size of the *Amaranthaceae* database, the *Mus musculus* sequences were added to ensure adequate database size for statistical scoring and calculation of false discovery rates (FDR) [38]. *Mus musculus* was chosen in order to avoid potential redundant homologous hits with a plant database and any mouse hits were thus ignored in data analysis. While there are EST databases available for *Beta vulgaris* [39,40], we found that there is no straightforward tool to convert the entire database/sequence information to a protein FASTA database. Due to additional complexities associated with assigning the intron/exon sites, start/stop codons, and a lack of meaningful protein annotation from these RNA databases we concluded that conversion would not be an accurate or effective way to identify putative proteins. The following search parameters were used in Mascot: monoisotopic mass, parent mass tolerance of 20 ppm, fragment ion mass tolerance of 0.8 Da, complete tryptic digestion allowing two missed cleavages, variable modification of methionine oxidation, and a fixed modification of cysteine carbamidomethylation. SEQUEST search parameters were the same except for a fragment ion mass tolerance of 1.0 Da and a parent ion tolerance of 0.0120 Da. Peptide identifications from both of the search engines were combined using probabilistic protein identification algorithms in Scaffold version 4.0.3 (Proteome Software, Portland, OR, USA) [41,42] with protein clustering enabled. All data files for each biological replicate were then combined using the “mudpit” option in Scaffold4 generating a composite listing for all proteins identified. Thresholds were set to 99% protein probability, 95% peptide probability, and a 2 unique peptide minimum was required. Peptides shared across proteins were apportioned between proteins according to a weighting function. The peptide FDR was 0% after manual validation of a subset of proteins identified by 2 unique peptides [43]. Criteria for manual validation included the following: (1) a minimum of at least 5 theoretical y or b ions in consecutive order that are peaks greater than 5% of the maximum intensity; (2) an absence of prominent unassigned peaks greater than 5% of the maximum intensity; and (3) indicative residue specific fragmentation, such as intense ions N-terminal to proline and immediately C-terminal to aspartate and glutamate. GO terms were then mapped by Scaffold4 using Uniprot and confirmed manually by literature review. All mass spectrometry data has been deposited to the ProteomeXchange [44] with identifier PXD000237.

### 2.4. Statistical Analyses for Differentially Expressed Proteins

Unweighted spectrum counts (SpC) and normalized quantitative value for total spectra and average total ion current (TIC) were exported from Scaffold4. *T*-tests were performed in Excel using the normalized quantitative value (for SpC and Avg TIC). Fold change was analyzed using the normalized quantitative value “plus 1” in Excel. Box plots were visualized in DanteR (Pacific Northwest National Laboratories) to assess the effect of normalization on the median spectral counts of each sample. Venn diagrams were generated in Scaffold4.

## 3. Results and Discussion

### 3.1. Protein Characterization

**Figure 1:**
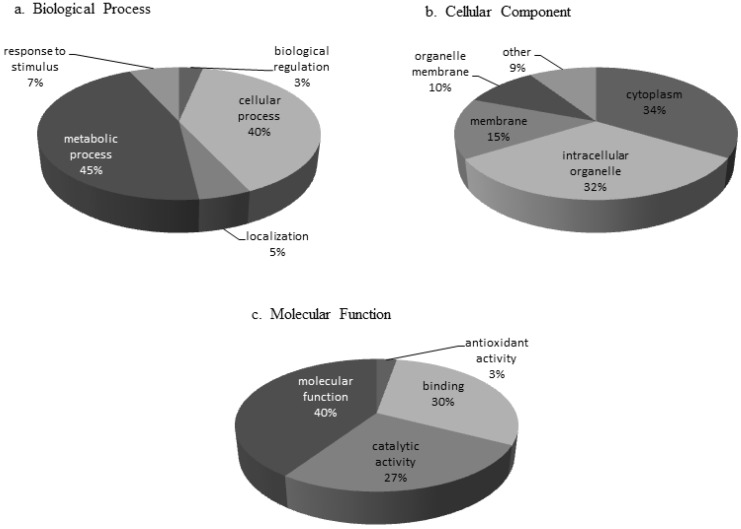
Gene Ontology (GO) terms for proteins identified from BNYVV infection of susceptible *Beta vulgaris* leaves. GO terms were collected by Scaffold4 from Uniprot and organized by (**a**) Biological Process (**b**) Cellular Component and (**c**) Molecular Function. Categories are represented as a percent of total identified GO terms.

**Figure 2:**
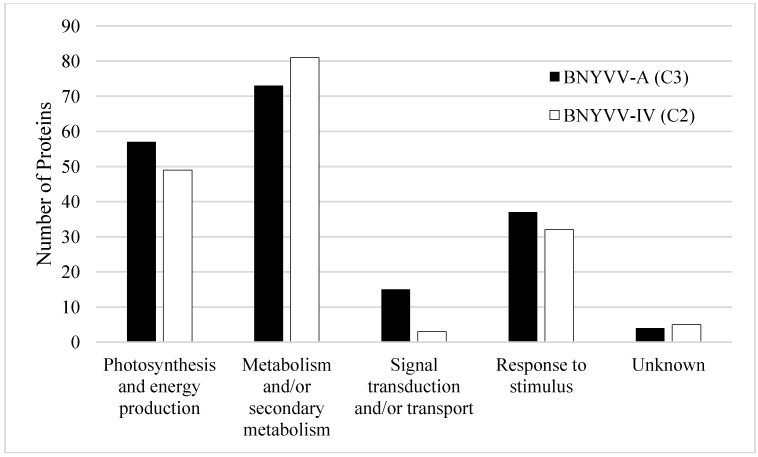
Total number of proteins identified from susceptible (R30_rz1) *Beta vulgaris* leaves during infection with the standard A-type (BNYVV-A) and the *Rz1* resistance-breaking strain (BNYVV-IV). Proteins are assigned to protein classes based on predicted annotated function as assigned by Scaffold4 using Uniprot and confirmed manually by literature review.

Prior to protein analysis the presence of BNYVV in test samples was confirmed by performing a BNYVV-specific ELISA assay on all pooled material. ELISA confirmed infection of the susceptible sugar beet variety by both BNYVV pathotypes, with a mean O.D. at 405 nm of 0.382 for BNYVV-IV, and 0.298 for BNYVV-A. Positive samples are considered those with O.D. values of at least twice that of healthy sugar beet (mean O.D. 0.145). Protein assignment was completed using Mascot analysis software and the Uniprot *Amaranthaceae* database with the accession number for each identified protein arranged by predicted annotated function (Supplementary Table S1). A total of 203 proteins (Supplementary Table S1) were identified that met our criteria as defined in the methodology, and mass spectrometry data has been deposited to the ProteomeXchange consortium [44] via the PRIDE partner repository [45] with the dataset identifier PXD000237. Normalized spectral abundance factors (NSAF) were calculated [46] (NSAF = (unweighted spectrum count/molecular weight)/sum spectral counts for experiment). The 203 proteins were categorized based on annotated GO terms for those proteins that had terms assigned within the categories biological process, cellular component, and molecular function (Figure 1). Categories with increased representation classified under biological process were predicted to function in metabolic (45%) and cellular processes (40%). The predominant protein classes under cellular components in leaf tissue were associated with cytoplasm (34%) and intracellular organelles (32%), while most proteins assigned under the molecular function category were classified as associated with molecular function (40%) and binding (30%) (Figure 1). The majority of the proteins found in the susceptible sugar variety were present during infection by both BNYVV pathotypes. However, there were unique proteins found in interactions with the standard A-type strain (BNYVV-A) that were not found in the resistance breaking strain (BNYVV-IV) and vice versa (Figure 2, Supplementary Table S1). Additionally, the total number of proteins in each predicted annotated function differed during each interaction. For example, there were more putative photosynthesis/energy production and metabolism proteins identified during the interaction with BNYVV-A than during interaction with the resistance breaking strain (BNYVV-IV). In contrast, more signal transduction and transport proteins were identified during the interaction with BNYVV-IV (Figure 2). This suggests that even in compatible interactions, different biological pathways are being induced to create a susceptible interaction in sugar beet dependent on the virus pathotype in question. In a previous study, Larson *et al.* [32] identified 50 proteins that were differentially up or down regulated in individual susceptible and resistant isogenic lines of sugar beet in response to BNYVV infection. In that study, the authors utilized a subtractive proteomic approach in which proteins were separated using a ProteomeLab PF2D, two-dimensional protein fractionation system (Beckman Coulter, Fullerton, CA, USA) and proteins were identified by MALDI-TOF/TOF mass spectrometry. Such an approach required a large amount of protein (>2 mg) in order to detect differential protein peaks for the subtractive analysis and therefore only those proteins with large differences and significant amounts of protein could be detected. In contrast, an untargeted, global proteomic profiling approach was used in our study, which allowed profiling of all detectable proteins, even those at low concentrations. This approach also allowed for the profiling of proteins expressed during compatible interactions with two pathotypes of BNYVV in a susceptible genotype. This global approach is critical, as it is important to understand all the potential metabolic pathways that may be induced during the response of sugar beet to pathogen infection and perhaps highlight the underlying plant defense response even in susceptible varieties, particularly because so little proteomic data is available for sugar beet. Importantly, this global LC-MS/MS approach is a significant improvement over the use of traditional 2D gels, allowing for easier sample preparation, method development, quantitation, and identification. Despite the difference in methodology, several proteins were identified in the present study that were also found by Larson *et al.* [32] (Supplementary Table S1) including glutamine synthetase, 50S ribosomal proteins, actin 1, profiling, and calmodulin, as well as several putative defense related proteins (superoxide dismutase, ascorbate peroxidase, peroxidases, and chitinase).

Many photosynthesis and energy related proteins were found to be expressed during compatible BNYVV interactions in sugar beet, including Ribulose bisphosphate(s), Chlorophyll a/b-binding proteins, photosystem reaction center subunits, ATPase(s), *etc.* (Supplementary Table S1). Management of energy resources by plants during periods of stress commonly occurs in order to provide the necessary energy required to mount a defense, and in some compatible virus/host interactions it is predicted that proteins involved in photosynthesis are likely activated by the virus itself, in order to produce the metabolic energy needed for viral replication (reviewed in [47]).

Primary metabolism in plants comprises all metabolic pathways that are essential to the plant’s survival, growth, and development. In contrast, secondary metabolites are compounds produced in other metabolic pathways that, although important, are not essential to the functioning of the plant; however, many of these proteins are important in plant defense. Many proteins identified in this study have been associated with primary and secondary metabolic processes in plants (Supplementary Table S1), including glutamine synthetase (B2CZA8) and carbonic anhydrase (P16016).

Signal transduction pathways, which transmit information within individual cells and throughout the plant, are activated whenever biotic and abiotic stresses are recognized at the cellular level, leading to changes in many pathways and cellular processes in plants. Several proteins were expressed during BNYVV infection that are predicted to be associated with signal transduction and transport including three proteins previously reported by Larson *et al.* [32] (Supplementary Table S1). One such protein, calmodulin, has been extensively studied and shown to play crucial role(s) in cellular signaling and regulation of numerous target proteins in plants (reviewed in [48]).

### 3.2. Response to Stimulus (Including Plant Defense Response) Proteins

Several proteins identified were of primary interest as they had been previously reported to be associated with plant defense responses or responses to other stimuli in plants. Some of these proteins include: glucan endo-1,3-β-D-glucosidase, a 14-3-3 like protein, a pathogenesis related protein, multiple peroxidases, and superoxide dismutase (Supplementary Table S1).

The glucan endo-1,3-β-D-glucosidase (Q9XFW8) is of particular interest. Proteins in this class have been implicated in the hydrolysis of 1,3-β-D-glucosidic linkages in 1,3-β-D-glucans [49] and may contribute to susceptibility of plants to viruses. In plants, callose (a 1,3-β-D-glucan [50,51]) is deposited between the plasma membrane and the cell wall during the hypersensitive response to infection by both fungi and viruses [49,52]. It is believed that callose acts as a physical barrier limiting the spread of pathogens in resistant plants [53]. It was previously shown that down-regulation of another closely related protein β-1,3-glucanase, contributes to restricting the cell-to-cell movement of TMV, reduced plasmodesmatal size exclusion limits, and enhanced callose deposition in plants [49,54]. How glucan endo-1,3-β-D-glucosidase (Q9XFW8) is “behaving” during the susceptible interaction of BNYVV with sugar beet is unknown, but its identification in this study opens up intriguing avenues of future research to characterize compatible BNYVV/sugar beet interactions.

Another protein of interest was identified as a putative 14-3-3-like protein (P29308). 14-3-3 proteins have been reported to bind other proteins in order to regulate their function in complex environmental signaling pathways and have long been thought to play a role in plant defenses against pathogens. A number of studies implicate some 14-3-3 proteins in R-gene mediated plant disease resistance (reviewed in [55,56]).

During pathogen infection various proteins are induced in association with development of systemic acquired resistance (SAR). These are collectively referred to as pathogenesis-related (PR) proteins. PR proteins are defined as host proteins that are induced by the plant after pathogen attack [57]. We only found a single PR protein (Pathogenesis-related protein 1a/B5QTD3) that was expressed during BNYVV compatible interactions, suggesting that although SAR may be induced in the compatible virus-host interactions, the host response may be limited. However, the specific nature and function of putative PR-1 protein family members is unclear and indicates that this generalized defense response should be evaluated further in in subsequent studies.

Three peroxidases were found to be expressed in compatible BNYVV interactions: ascorbate peroxide (Q42459), and two peroxidases (P93552 and P93547). Peroxidases have been reported to play roles in plant defense to pathogens as a means of protection against the increased production of reactive oxygen species during the hypersensitive response and systemic acquired resistance pathways [58,59,60]. These have been correlated with accumulation of key enzymes important for building plant cell walls in resistant plants [58]. How putative peroxidases are being utilized in sugar beet during compatible interactions is unknown but they could be associated with generalized plant defense responses to BNYVV infection.

Superoxide dismutases are metalloenzymes found in plants and other organisms that protect cells against super-oxide radicals and prevent formation of other active species of oxygen. During the plant defense response superoxide dismutase expression increases, leading to generation of hydrogen peroxide and increased tolerance to pathogens [61,62]. Up-regulation of superoxide dismutase is believed to contribute in preventing oxidative damage in plants under stress [61,62]. A superoxide dismutase (A7WTB6) was expressed in both compatible BNYVV reactions in the susceptible sugar beet variety lending substantial support to the activity of this enzyme during BNYVV infection; how this protein is contributing to plant defense during compatible interactions should be investigated in future experiments.

### 3.3. Differential Expression of Proteins during Compatible BNYVV Interactions

During initial proteomic profiling we found that, in general, most of the proteins identified were found during interactions with both BNYVV pathotypes (Figure 2). Using statistical analysis of the unweighted spectrum counts (SpC) and average total ion current (Avg TIC) we found that eight proteins were more highly expressed during infection with the standard A-type strain (BNYVV-A) compared with the resistant breaking strain (BNYVV-IV) (Table 1). All eight proteins were identified in both interactions (Supplementary Table S1) but here we show that some proteins were more highly expressed. The proteins found to be differentially expressed during infection by the standard A-type strain generally fell into the categories metabolism/secondary metabolism and photosynthesis and energy production. Additionally, two proteins were identified that were previously found to be associated with stress responses in plants; a superoxide dismutase and a cytosolic heat shock protein.

**Table 1:** Proteins differentially expressed in a susceptible sugar beet variety by a standard A-type strain (BNYVV-A) compared to infection by the *Rz1* resistance-breaking strain (BNYVV-IV).

Protein Name	Accession Number	Spectral Counting *p*-value (*p* < 0.1) ^1^	Average TIC *p*-value (*p* < 0.1) ^2^
Photosystem II CP43 chlorophyll apoprotein	PSBC_SPIOL	0.025	0.001
Cytosolic heat shock 70 protein	Q41374_SPIOL	0.053	
Superoxide dismutase [Cu-Zn]	H9BQP7_SUASA	0.100	
Glyceraldehyde-3-phosphate dehydrogenase B, chloroplastic	G3PB_SPIOL		0.010
Choline monooxygenase, chloroplastic	CHMO_BETVU		0.032
Fructose-bisphosphate aldolase, chloroplastic	ALFC_SPIOL		0.033
Dehydroascorbate reductase	Q9FVE4_SPIOL		0.061
Formate—tetrahydrofolate ligase	FTHS_SPIOL		0.061

^1^ Spectral counting compares the total number of peptide spectra that match to a protein between two groups. ^2^ Average TIC measures the differences in average total ion current off of the mass spectrometer between the two samples.

Pathogen fitness is the ability of the organism to survive and reproduce [63] but can be defined further by using many factors including the level of aggressiveness of the pathogen in susceptible hosts, the ability of the pathogen to survive or persist among a population of other variants or other pathogens, the ability of the pathogen to multiply and spread within the host, as well as having the ability to spread to new hosts ([64], reviewed in [65]). It has been shown that mutations that allow a pathogen to overcome particular resistance genes (*i.e.*, loss of avirulence) also incur a fitness cost to the pathogen during compatible interactions which include decreased aggressiveness (the amount of disease) and reduced symptom expression (reviewed in [65]). Therefore, in susceptible plants, it is possible that the mutation that allows a resistant breaking pathotype to cause disease on resistant hosts, also changes how the pathogen interacts with susceptible hosts and likewise the molecular pathways that are activated in the susceptible host. The finding that there were eight proteins that are more highly expressed in a compatible interaction with the standard A-type BNYVV strain compared with the resistance breaking strain (BNYVV-IV) suggests that a fitness cost may have been incurred in the resistance breaking strain. Characterization of these mechanisms and their role in pathogen fitness should be more fully elucidated in future experiments.

## 4. Conclusions

Mass spectrometry based proteomic investigations of non-model systems, such as sugar beet, are challenging due to the lack of a well annotated sequenced genome. Larson *et al.* [32] were only able to identify ~42% of the differentially expressed proteins in that study due to reliance on homology with closely related, fully sequenced organisms when performing database searches for protein identification. A complete genomic sequence for sugar beet was only recently released [66] however it has neither been translated into a protein FASTA database, nor fully annotated. This greatly limits its utility for proteomic applications. The study presented herein represents the first large scale shotgun proteomic analysis of sugar beet, which, combined with homology based database searching against the Uniprot *Amaranthaceae* protein sequence database yielded the confident identification of 203 proteins from BNYVV infected sugar beet. Many proteins identified in susceptible sugar beet during infection with BNYVV were expressed with not just one pathotype, but both. These two BNYVV pathotypes represent the known diversity of BNYVV in the United States (with regards to performance against resistant sources), which justifies future downstream studies to determine specific pathways that may be involved in BNYVV pathogenesis. Understanding how sugar beet responds to infection by BNYVV will enable researchers and producers to identify more effective or novel methods for control of BNYVV infection and rhizomania disease through biotechnology, and may result in improved approaches to manage resistant germplasm, and potentially prolong the functional life of the *Rz* set of resistance genes. The results of this study lay the groundwork for expanded research into the mechanisms and pathways of compatible interactions between BNYVV and sugar beet that lead to development of rhizomania disease. Using the methods established herein, we plan future studies to describe the quantitative differences in protein expression between defined treatment groups. Such a large-scale quantitative approach will be the first of its kind in the field of sugar beet proteomics and should provide critical information on interactions between a virus and disease resistance genes.

## Supplementary Materials

Supplementary File 1
