# Supplementary material for: Proteomic Profiling of Sugar Beet (Beta vulgaris) Leaves during Rhizomania Compatible Interactions
Source: Proteomes. 2014 Apr 9;2(2):208–23. doi: 10.3390/proteomes2020208 (PMC5302737; doi:10.3390/proteomes2020208)
Supplement: Supplementary File 1 [file proteomes-02-00208-s001.pdf]

# Supplementary Material

**Table S1.** List of proteins identified in the *Beta vulgaris* leaf proteome during BNYVV infection of susceptible sugar beet via mass spectrometry.

| Protein Name [1] | Uniprot Accession | Name | Mass kDa | NSAF [2] | Number Unique Peptides | % Coverage | BNYVV-IV (C2) [3] | BNYVV-IV (C3) [4] |
|---|---|---|---|---|---|---|---|---|
| **Photosynthesis and energy production** | | | | | | | | |
| Ribulose bisphosphate carboxylase large chain | Q4PLI7 | rbcL | 53 | 3.50E−03 | 88 | 66% | x | x |
| ATP synthase subunit beta, chloroplastic | Q4PLI6 | atpB | 54 | 1.40E−03 | 39 | 85% | x | x |
| Ribulose bisphosphate carboxylase small chain | P00870 | RBCS | 14 | 1.00E−03 | 6 | 51% | x | x |
| Rubisco activase | Q8L5T3 | rca | 48 | 7.80E−04 | 25 | 49% | x | x |
| ATP synthase subunit alpha, chloroplastic | P06450 | atpA | 55 | 6.20E−04 | 35 | 47% | x | x |
| Cytochrome b559 subunit alpha | Q2Z1Q5 | psbE | 9 | 4.00E−04 | 4 | 48% | x | x |
| Photosystem I iron-sulfur center | P10098 | psaC | 9 | 3.60E−04 | 8 | 83% | x | x |
| Photosystem I reaction center subunit IV, chloroplastic | P12354 | PSAE-1 | 13 | 3.50E−04 | 4 | 35% | x | x |
| Glyceraldehyde-3-phosphate dehydrogenase B, chloroplastic | P12860 | GAPB | 48 | 3.30E−04 | 10 | 33% | x | x |
| Photosystem I reaction center subunit II, chloroplastic | P12353 | psaD | 23 | 3.20E−04 | 9 | 32% | x | x |
| Phosphoglycerate kinase, chloroplastic | P29409 | | 46 | 3.10E−04 | 16 | 42% | x | x |
| Chlorophyll a/b-binding protein | O49812 | | 28 | 3.10E−04 | 9 | 57% | x | x |
| Phosphoribulokinase, chloroplastic | P09559 | | 45 | 2.90E−04 | 5 | 46% | x | x |
| Glycine decarboxylase subunit T | Q947L6 | gdt | 14 | 2.80E−04 | 7 | 78% | x | x |
| Glyceraldehyde-3-phosphate dehydrogenase A, chloroplastic | P19866 | GAPA | 43 | 2.70E−04 | 8 | 28% | x | x |
| Oxygen-evolving enhancer protein 1, chloroplastic | P12359 | PSBO | 35 | 2.60E−04 | 2 | 25% | | x |
| ATP synthase subunit b, chloroplastic | P06453 | atpF | 21 | 2.00E−04 | 7 | 38% | x | x |
| 23 kDa OEC protein | B0L802 | PsbP | 22 | 1.70E−04 | 4 | 15% | x | x |

**Table S1.** *Cont.*

| Protein Name [1] | Uniprot Accession | Name | Mass kDa | NSAF [2] | Number Unique Peptides | % Coverage | BNYVV-IV (C2) [3] | BNYVV-IV (C3) [4] |
|---|---|---|---|---|---|---|---|---|
| Photosystem I reaction center subunit VI, chloroplastic | P22179 | PSAH | 15 | 1.60E−04 | 2 | 8.30% | x | x |
| Dihydrolipoamide dehydrogenase | Q947M1 | dhl | 18 | 1.60E−04 | 6 | 51% | x | x |
| V-type proton ATPase catalytic subunit A | Q39442 | | 69 | 1.60E−04 | 27 | 54% | x | x |
| Chlorophyll a/b binding protein | Q7XAC6 | cab11 | 28 | 1.40E−04 | 7 | 52% | x | x |
| Sedoheptulose-1,7-bisphosphatase, chloroplastic | O20252 | | 42 | 1.20E−04 | 7 | 19% | x | x |
| Photosynthetic oxygen-evolving protein 16 kDa subunit | B0L6Y3 | psbQ | 25 | 1.20E−04 | 2 | 15% | x | x |
| Apocytochrome f | P16013 | petA | 35 | 1.10E−04 | 7 | 20% | x | x |
| Glycine decarboxylase subunit P | Q947L7 | gdp | 8 | 1.00E−04 | 5 | 40% | | x |
| Photosystem II reaction center protein H | P05146 | psbH | 8 | 9.30E−05 | 2 | 29% | x | x |
| Cytochrome b6 | P00165 | petB | 24 | 9.20E−05 | 3 | 21% | x | x |
| Triosephosphate isomerase, chloroplastic | P48496 | TPIP1 | 34 | 8.90E−05 | 6 | 32% | x | x |
| Chloroplast ribose-5-phosphate isomerase | Q8RU73 | | 31 | 8.80E−05 | 4 | 16% | x | x |
| ADP-ribosylation factor 1 | H2EP74 | ARF1 | 21 | 8.50E−05 | 8 | 46% | x | x |
| Fructose-1,6-bisphosphatase, chloroplastic | P22418 | | 45 | 8.10E−05 | 4 | 13% | x | x |
| ATP-dependent Clp protease proteolytic subunit | Q9M3K5 | clpP | 22 | 6.90E−05 | 2 | 21% | | x |
| ATP synthase subunit b', chloroplastic | P31853 | ATPG | 24 | 6.40E−05 | 2 | 9.00% | x | x |
| ATP synthase epsilon chain, chloroplastic | P00833 | atpE | 15 | 5.90E−05 | 2 | 16% | x | x |
| ATP synthase delta chain, chloroplastic | P11402 | ATPD | 28 | 5.70E−05 | 3 | 7.40% | | x |
| Photosystem I reaction center subunit XI, chloroplastic | Q41385 | PSAL | 23 | 5.40E−05 | 2 | 12% | x | x |

**Table S1.** *Cont.*

| Protein Name [1] | Uniprot Accession | Name | Mass kDa | NSAF [2] | Number Unique Peptides | % Coverage | BNYVV-IV (C2) [3] | BNYVV-IV (C3) [4] |
|---|---|---|---|---|---|---|---|---|
| V-type proton ATPase subunit E | Q41396 | VATE | 26 | 5.40E−05 | 9 | 48% | x | x |
| Photosystem II 22 kDa protein, chloroplastic | Q02060 | PSBS | 29 | 4.70E−05 | 4 | 20% | x | x |
| Photosystem II CP43 chlorophyll apoprotein | Q6EY72 | psbC | 46 | 4.60E−05 | 2 | 19% | x | x |
| Photosystem I reaction center subunit V, chloroplastic | P12357 | PSAG | 18 | 4.50E−05 | 3 | 10% | x | x |
| Ribulose-phosphate 3-epimerase, chloroplastic | Q43157 | RPE | 30 | 4.50E−05 | 2 | 15% | | x |
| Photosystem II D2 protein | P06005 | psbD | 40 | 4.10E−05 | 3 | 9.10% | x | x |
| Cytochrome b6-f complex iron-sulfur subunit, chloroplastic | P08980 | petC | 24 | 4.00E−05 | 3 | 17% | x | x |
| Photosystem II CP43 chlorophyll apoprotein | P06003 | psbC | 52 | 3.60E−05 | 3 | 25% | x | x |
| Photosystem I reaction center subunit III, chloroplastic | P12355 | PSAF | 25 | 3.50E−05 | 4 | 20% | x | x |
| Photosystem Q(B) protein | P69565 | psbA | 39 | 1.90E−05 | 4 | 16% | x | x |
| Delta-aminolevulinic acid dehydratase, chloroplastic | P24493 | HEMB | 47 | 1.90E−05 | 3 | 18% | x | x |
| Photosystem I P700 chlorophyll a apoprotein A2 | P06512 | psaB | 82 | 1.90E−05 | 5 | 9.80% | x | x |
| Oxygen-evolving enhancer protein 3, chloroplastic | P12301 | PSBQ | 25 | 1.70E−05 | 3 | 5.20% | x | x |
| Vacuolar proton pump ATPase subunit H | G4XKY2 | VHA-H | 53 | 1.50E−05 | 5 | 12% | x | x |
| Violaxanthin de-epoxidase, chloroplastic | Q9SM43 | VDE1 | 54 | 1.40E−05 | 3 | 7.80% | x | x |
| Type III chlorophyll a/b-binding protein | Q9AVE9 | cab3 | 17 | 9.90E−06 | 2 | 16% | | x |
| NADPH-protochlorophyllide oxidoreductase 2 | Q9AVF1 | POR2 | 25 | 4.80E−06 | 3 | 20% | | x |
| UDP-glucose:flavonoid-O-glucosyltransferase | Q5GIG8 | | 54 | 4.00E−06 | 2 | 6.50% | x | |

**Table S1.** *Cont.*

| Protein Name [1] | Uniprot Accession | Name | Mass kDa | NSAF [2] | Number Unique Peptides | % Coverage | BNYVV-IV (C2) [3] | BNYVV-IV (C3) [4] |
|---|---|---|---|---|---|---|---|---|
| 26S protease regulatory subunit 7 | Q41365 | RPT1 | 48 | 2.00E−06 | 2 | 6.60% | | x |
| Photosystem I P700 chlorophyll a apoprotein A1 | P06511 | psaA | 83 | 1.70E−06 | 2 | 2.30% | | x |
| **Metabolism and/or secondary metabolism** | | | | | | | | |
| Glyceraldehyde-3-phosphate dehydogenase | A3FMH0 | | 37 | 5.10E−04 | 19 | 79% | x | x |
| **Glutamine synthetase** | **B2CZA8** | **GNL2** | **47** | **4.00E−04** | **20** | **56%** | **x** | **x** |
| Malate dehydrogenase, cytoplasmic | Q9SML8 | NR1 | 35 | 3.60E−04 | 16 | 64% | x | x |
| Serine hydroxymethyltransferase | Q947K8 | sht | 5 | 2.70E−04 | 2 | 32% | x | x |
| Fructose-bisphosphate aldolase, chloroplastic | P16096 | | 42 | 2.50E−04 | 13 | 32% | x | x |
| Methionine synthase | Q4H1G2 | BvMS1 | 88 | 2.30E−04 | 15 | 48% | x | x |
| **50S ribosomal protein L12, chloroplastic** | **Q53WU1** | **Soc** | **20** | **2.30E−04** | **6** | **33%** | **x** | **x** |
| Alpha tubulin | B5BT09 | TUB | 24 | 2.00E−04 | 6 | 56% | x | x |
| S-adenosylmethionine synthase 1 | Q4H1G4 | SAMS1 | 43 | 1.80E−04 | 22 | 68% | x | x |
| Adenosylhomocysteinase | Q4H1G1 | BvSAHH1 | 53 | 1.70E−04 | 24 | 60% | x | x |
| Transketolase, chloroplastic | O20250 | | 80 | 1.70E−04 | 13 | 21% | x | x |
| ClpC protease | O98447 | clpC | 99 | 1.60E−04 | 39 | 51% | x | x |
| Elongation factor 1-alpha | Q8H9A9 | skef-1A | 49 | 1.60E−04 | 19 | 38% | x | x |
| 50S ribosomal protein L14, chloroplastic | P09596 | rpl14 | 13 | 1.30E−04 | 5 | 51% | x | x |
| Carbonic anhydrase, chloroplastic | P16016 | | 35 | 1.10E−04 | 3 | 13% | x | x |
| Elongation factor 2 | O23755 | | 94 | 1.10E−04 | 28 | 50% | x | x |
| Sal k 3 pollen allergen | C1KEU0 | | 84 | 1.10E−04 | 2 | 22% | x | x |
| Choline monooxygenase, chloroplastic | O22553 | CMO | 50 | 1.00E−04 | 12 | 33% | x | x |
| Elongation factor 1-delta | O81918 | | 25 | 1.00E−04 | 5 | 40% | x | x |
| **Glutamate synthase** | **I6PD11** | | **157** | **9.60E−05** | **46** | **42%** | **x** | **x** |
| Carbonic anhydrase | Q947M3 | | 14 | 9.30E−05 | 2 | 20% | | x |
| 30S ribosomal protein S17, chloroplastic | P82137 | RPS17 | 4 | 9.00E−05 | 2 | 47% | | x |
| Enolase | Q9LEE0 | eno | 48 | 8.40E−05 | 9 | 29% | x | x |
| Fructose-bisphosphate aldolase | Q6RSN7 | | 39 | 7.10E−05 | 6 | 25% | x | x |

**Table S1.** *Cont.*

| Protein Name [1] | Uniprot Accession | Name | Mass kDa | NSAF [2] | Number Unique Peptides | % Coverage | BNYVV-IV (C2) [3] | BNYVV-IV (C3) [4] |
|---|---|---|---|---|---|---|---|---|
| Glutamate 1-semialdehyde aminotransferase | Q9AVF5 | GSA-AT | 33 | 7.10E−05 | 6 | 23% | x | x |
| Glucose-1-phosphate adenylyltransferase small subunit, chloroplastic/amyloplastic | P55232 | AGPB1 | 54 | 6.60E−05 | 15 | 43% | x | x |
| 30S ribosomal protein S11, chloroplastic | P06506 | rps11 | 15 | 6.60E−05 | 3 | 24% | x | x |
| Pyruvate dehydrogenase E1alpha subunit | Q852S0 | PDH E1a-1 | 44 | 6.50E−05 | 8 | 29% | x | x |
| 30S ribosomal protein S4, chloroplastic | P13788 | rps4 | 23 | 6.10E−05 | 8 | 39% | x | x |
| 50S ribosomal protein L5, chloroplastic | P82192 | RPL5 | 24 | 6.00E−05 | 2 | 12% | x | x |
| Plasma membrane intrinsic protein PIP1;1 | C7DYC4 | | 31 | 5.80E−05 | 6 | 36% | x | x |
| 30S ribosomal protein S16, chloroplastic | P28807 | rps16 | 10 | 5.80E−05 | 2 | 24% | x | x |
| 50S ribosomal protein L2, chloroplastic | P06509 | rpl2-A | 30 | 5.60E−05 | 5 | 25% | x | x |
| Nucleoside diphosphate kinase 2, chloroplastic | Q01402 | NDPK2 | 26 | 5.20E−05 | 3 | 15% | x | x |
| 30S ribosomal protein S1, chloroplastic | P29344 | RPS1 | 45 | 5.10E−05 | 8 | 17% | x | x |
| Fructose-1,6-bisphosphatase, cytosolic | Q42649 | | 37 | 5.00E−05 | 3 | 25% | x | x |
| Isocitrate dehydrogenase | Q9SPH8 | Icdh | 28 | 4.80E−05 | 3 | 12% | x | x |
| Uroporphyrinogen decarboxylase | Q9AVF8 | UROD | 21 | 4.70E−05 | 4 | 26% | x | x |
| Proteasome subunit alpha type | I6U5E4 | PAC1 | 27 | 4.60E−05 | 2 | 28% | x | x |
| Ferredoxin—NADP reductase, chloroplastic | P00455 | PETH | 41 | 4.60E−05 | 4 | 13% | x | x |
| Fructokinase | Q42645 | | 35 | 4.10E−05 | 10 | 40% | x | x |
| Cysteine synthase | B5U9U9 | CSase A | 34 | 4.10E−05 | 4 | 20% | x | x |
| Nitrite reductase | E2JFE1 | NiR | 67 | 4.00E−05 | 8 | 30% | x | x |
| Glutamine synthetase | B0LSR3 | GNL1 | 39 | 3.90E−05 | 6 | 32% | x | x |
| Ribosome-recycling factor, chloroplastic | P82231 | RRF | 30 | 3.50E−05 | 4 | 13% | x | x |
| Ketol-acid reductoisomerase, chloroplastic | Q01292 | AHRI | 64 | 3.50E−05 | 11 | 24% | x | x |

**Table S1.** *Cont.*

| Protein Name [1] | Uniprot Accession | Name | Mass kDa | NSAF [2] | Number Unique Peptides | % Coverage | BNYVV-IV (C2) [3] | BNYVV-IV (C3) [4] |
|---|---|---|---|---|---|---|---|---|
| Fructose-bisphosphate aldolase, cytoplasmic isozyme | P29356 | | 38 | 3.30E−05 | 3 | 9.50% | x | x |
| Dehydroascorbate reductase | Q9FVE4 | DHAR | 30 | 3.30E−05 | 3 | 11% | x | x |
| 30S ribosomal protein S13, chloroplastic | P82163 | RPS13 | 16 | 3.20E−05 | 2 | 9.70% | x | x |
| 40S ribosomal protein S25 | Q94G66 | RPS25 | 13 | 2.80E−05 | 2 | 21% | x | x |
| 60S acidic ribosomal protein P0 | P29764 | | 34 | 2.70E−05 | 3 | 8.70% | x | x |
| 30S ribosomal protein S9, chloroplastic | P82278 | PRPS9 | 21 | 2.60E−05 | 3 | 23% | x | x |
| Peptidyl-prolyl cis-trans isomerase, chloroplastic | O49939 | TLP40 | 50 | 2.40E−05 | 3 | 10% | x | x |
| 30S ribosomal protein S5, chloroplastic | Q9ST69 | rps5 | 34 | 2.40E−05 | 3 | 13% | x | x |
| Acyl-[acyl-carrier-protein] desaturase, chloroplastic | P28645 | | 46 | 2.30E−05 | 4 | 13% | x | x |
| 50S ribosomal protein L24, chloroplastic | P27683 | RPL24 | 21 | 2.30E−05 | 2 | 10% | x | |
| Formate—tetrahydrofolate ligase | P28723 | | 68 | 2.30E−05 | 6 | 14% | x | x |
| 30S ribosomal protein S8, chloroplastic | P09597 | rps8 | 16 | 2.10E−05 | 3 | 22% | x | x |
| rRNA N-glycosidase | B1VCR4 | | 28 | 2.00E−05 | 3 | 14% | x | x |
| 50S ribosomal protein L1, chloroplastic | Q9LE95 | RPL1 | 39 | 2.00E−05 | 2 | 6.80% | x | x |
| Phosphoenolpyruvate carboxylase | B2MW80 | | 110 | 1.90E−05 | 16 | 15% | x | x |
| Phosphoethanolamine N-methyltransferase | Q9M571 | PEAMT | 56 | 1.70E−05 | 4 | 8.30% | | x |
| Coproporphyrinogen oxidase | Q9AVF7 | COPROX | 19 | 1.60E−05 | 2 | 12% | | x |
| Sucrose synthase | Q6SJP5 | SBSS2 | 92 | 1.60E−05 | 7 | 16% | x | x |
| 40S ribosomal protein S11 | Q1H8R1 | rpS11 | 18 | 1.60E−05 | 2 | 15% | x | x |
| Fatty acid hydroperoxide lyase | E7E818 | HPL | 55 | 1.40E−05 | 5 | 13% | x | x |
| 50S ribosomal protein L16, chloroplastic | P17353 | rpl16 | 15 | 1.10E−05 | 2 | 16% | x | x |

**Table S1.** *Cont.*

| Protein Name [1] | Uniprot Accession | Name | Mass kDa | NSAF [2] | Number Unique Peptides | % Coverage | BNYVV-IV (C2) [3] | BNYVV-IV (C3) [4] |
|---|---|---|---|---|---|---|---|---|
| Granule bound starch synthase I | D6RSA2 | GBSSI | 67 | 1.10E−05 | 3 | 6.90% | x | |
| Malate dehydrogenase [NADP], chloroplastic | P52426 | MDH | 47 | 1.00E−05 | 2 | 6.40% | | x |
| Proteasome subunit beta type-5 | O24361 | | 30 | 9.60E−06 | 2 | 8.50% | x | |
| 30S ribosomal protein S19 alpha, chloroplastic | P06508 | rps19 | 11 | 8.70E−06 | 2 | 33% | | x |
| Phosphoserine aminotransferase, chloroplastic | P52877 | | 47 | 8.70E−06 | 4 | 10.00% | x | x |
| 30S ribosomal protein S3, chloroplastic | P09595 | rps3 | 25 | 8.60E−06 | 5 | 23% | | x |
| 6-phosphogluconate dehydrogenase, decarboxylating 1 | Q94KU1 | pgdC | 53 | 6.80E−06 | 3 | 12% | x | x |
| 50S ribosomal protein L11, chloroplastic | P31164 | rpl11 | 24 | 6.00E−06 | 2 | 14% | x | x |
| 11S globulin seed storage protein | Q38712 | | 57 | 5.90E−06 | 2 | 4.40% | x | x |
| Putative vacuolar processing enzyme | Q949L7 | | 54 | 5.80E−06 | 3 | 8.60% | x | |
| UDP-sulfoquinovose synthase, chloroplastic | Q84KI6 | SQD1 | 54 | 5.80E−06 | 2 | 7.70% | x | x |
| Delta 1-pyrroline-5-carboxylate synthetase | Q8LRT0 | P5CS | 24 | 4.00E−06 | 2 | 15% | | x |
| Monodehydroascorbate reductase | Q94IB7 | | 54 | 4.00E−06 | 2 | 6.40% | x | x |
| Myo-inositol-1-phosphate synthase | Q944C3 | INPS | 57 | 3.80E−06 | 2 | 6.10% | | x |
| Hexokinase-1 | Q9SEK3 | HXK1 | 54 | 3.60E−06 | 2 | 8.00% | | x |
| Cysteine synthase like protein | Q767A2 | CSaseLP | 36 | 3.30E−06 | 2 | 11% | | x |
| Glucose-6-phosphate isomerase | O82058 | GPIP | 68 | 2.80E−06 | 2 | 4.00% | x | x |
| Glycylpeptide N-tetradecanoyltransferase | K4Q1D5 | | 51 | 1.90E−06 | 2 | 7.80% | | x |
| Glucose-1-phosphate adenylyltransferase large subunit, chloroplastic/amyloplastic | P55233 | AGPS1 | 58 | 1.70E−06 | 2 | 4.00% | x | x |

**Table S1.** *Cont.*

| Protein Name [1] | Uniprot Accession | Name | Mass kDa | NSAF [2] | Number Unique Peptides | % Coverage | BNYVV-IV (C2) [3] | BNYVV-IV (C3) [4] |
|---|---|---|---|---|---|---|---|---|
| **Signal transduction and/or transport** | | | | | | | | |
| 33 kDa protein of the oxygen-evolving complex | B5BT06 | OEE1 | 35 | 4.70E−04 | 7 | 56% | x | x |
| Non-specific lipid-transfer protein | Q7XZE0 | AnLTP | 12 | 4.30E−04 | 2 | 11% | x | |
| Beta-tubulin | A3FMG7 | | 12 | 3.50E−04 | 6 | 60% | x | |
| **Actin 1** | **E9P160** | | **42** | **3.50E−04** | **20** | **64%** | **x** | |
| **Profilin** | **A8VT60** | | **14** | **2.20E−04** | **3** | **24%** | **x** | |
| **Calmodulin** | **Q3UKW2** | **Calm1** | **22** | **1.50E−04** | **7** | **73%** | **x** | |
| Voltage-dependent anion channel protein | Q41368 | SVDAC1 | 30 | 1.50E−04 | 7 | 30% | x | |
| 28 kDa ribonucleoprotein, chloroplastic | P28644 | | 25 | 1.30E−04 | 6 | 30% | x | |
| Putative GTP binding protein | Q0MX58 | | 23 | 9.40E−05 | 7 | 27% | x | |
| Luminal-binding protein | Q42434 | HSC70 | 74 | 5.50E−05 | 11 | 26% | x | |
| Plasma membrane major intrinsic protein 2 | Q39440 | | 30 | 5.00E−05 | 9 | 41% | x | |
| 20 kDa chaperonin, chloroplastic | Q02073 | CPN21 | 27 | 4.80E−05 | 2 | 7.10% | x | |
| Calreticulin | O81919 | | 48 | 4.00E−05 | 7 | 29% | x | |
| Chloroplast mRNA-binding protein CSP41 | O24365 | | 45 | 2.70E−05 | 3 | 6.70% | x | |
| Protein phosphatase 2A | H6VX93 | PP2A | 35 | 8.20E−06 | 3 | 21% | | x |
| Protein translocase subunit SecA, chloroplastic | Q36795 | secA | 117 | 4.70E−06 | 2 | 3.40% | | x |
| **Response to stimulus (including plant defense response)** | | | | | | | | |
| Peroxisomal (S)-2-hydroxy-acid oxidase | P05414 | | 40 | 3.40E−04 | 17 | 54% | x | x |
| Cytosolic heat shock 70 protein | O22664 | HSC70 | 71 | 2.40E−04 | 25 | 45% | x | x |
| Peroxiredoxin Q, chloroplastic | Q6UBI3 | PRXQ | 24 | 2.30E−04 | 8 | 36% | x | x |
| **Superoxide dismutase [Cu-Zn]** | **A7WTB6** | | **22** | **2.20E−04** | **6** | **20%** | **x** | **x** |
| Heat shock 70 protein | O50036 | HSC70-9 | 76 | 1.90E−04 | 24 | 38% | x | x |
| Glucan endo-1,3-beta-D-glucosidase | Q9XFW8 | Glu2 | 36 | 1.50E−04 | 14 | 63% | x | x |
| 14-3-3-like protein | P29308 | | 25 | 1.40E−04 | 9 | 28% | x | x |
| **Ascorbate peroxidase** | **Q42459** | | **28** | **1.30E−04** | **3** | **28%** | **x** | **x** |

**Table S1.** *Cont.*

| Protein Name [1] | Uniprot Accession | Name | Mass kDa | NSAF [2] | Number Unique Peptides | % Coverage | BNYVV-IV (C2) [3] | BNYVV-IV (C3) [4] |
|---|---|---|---|---|---|---|---|---|
| Catalase | Q94EV9 | CAT1 | 57 | 1.30E−04 | 9 | 23% | x | x |
| 2-Cys peroxiredoxin BAS1, chloroplastic | O24364 | BAS1 | 29 | 1.30E−04 | 5 | 25% | x | x |
| Cell wall peroxidase | O81266 | | 9 | 7.70E−05 | 2 | 61% | x | x |
| Laccase-like protein | I3W7E6 | | 51 | 7.50E−05 | 12 | 36% | x | x |
| Superoxide dismutase | H9BQP5 | MnSOD | 26 | 7.50E−05 | 4 | 25% | x | x |
| Thioredoxin M-type, chloroplastic | P07591 | | 20 | 7.40E−05 | 3 | 10% | x | x |
| Thaumatin like protein | Q949L8 | | 23 | 5.70E−05 | 3 | 20% | x | x |
| Peroxidase prx13 | Q9M4Z4 | | 36 | 5.40E−05 | 3 | 17% | x | x |
| **Chitinase** | **Q9XFW7** | **Ch4** | **28** | **5.10E−05** | **7** | **44%** | **x** | **x** |
| Heat shock protein 83 | Q1H8M1 | hsp83 | 40 | 4.60E−05 | 7 | 28% | x | x |
| Germin-like protein Kiel 1 | Q84RC0 | GLP-Ki1 | 22 | 4.40E−05 | 3 | 11% | x | x |
| Pathogenesis-related protein 1a | B5QTD3 | pr1a | 18 | 3.90E−05 | 3 | 32% | x | |
| Germin-like protein Wageningen 1 | Q84V63 | GLP-Wag1 | 22 | 3.50E−05 | 4 | 16% | x | |
| Betaine aldehyde dehydrogenase, chloroplastic | P28237 | | 55 | 3.40E−05 | 12 | 29% | x | x |
| Salt tolerance protein 4 | Q84LL8 | sato4 | 22 | 2.90E−05 | 2 | 16% | x | x |
| Salt tolerance protein 2 | Q711N3 | sato2 | 38 | 2.70E−05 | 6 | 23% | x | x |
| Major latex like protein homolog | Q949M0 | | 17 | 2.40E−05 | 4 | 27% | x | x |
| Peroxisomal ascorbate peroxidase | L0CTS4 | | 31 | 2.30E−05 | 6 | 31% | x | x |
| **Peroxidase** | **P93552** | **prxr8** | **36** | **2.20E−05** | **3** | **15%** | **x** | |
| Catalase | Q941J0 | cat2 | 33 | 2.00E−05 | 3 | 12% | x | x |
| Heat shock 70 protein | O49045 | HSC70-10 | 72 | 1.90E−05 | 5 | 16% | x | x |
| **Peroxidase** | **P93547** | **prxr3** | **38** | **1.80E−05** | **2** | **11%** | **x** | |
| Acidic endochitinase SP2 | P42820 | SP2 | 30 | 1.70E−05 | 5 | 24% | x | |
| Salt tolerance protein 5 | Q84LL6 | sato5 | 33 | 1.50E−05 | 2 | 17% | x | x |
| Auxin-induced beta-glucosidase | Q7XJH8 | | 83 | 1.40E−05 | 4 | 8.00% | x | x |
| Acidic endochitinase SE2 | P36910 | SE2 | 31 | 1.40E−05 | 2 | 11% | x | |
| Cationic peroxidase | Q1H8N1 | cprx1 | 35 | 1.00E−05 | 4 | 25% | x | x |
| DnaJ protein homolog ANJ1 | P43644 | | 47 | 9.70E−06 | 2 | 8.90% | x | x |
| **Chitinase** | **Q42421** | **Ch1** | **48** | **6.50E−06** | **3** | **13%** | **x** | **x** |
| Stress-induced protein sti1-like protein | H9B3R7 | | 51 | 1.40E−06 | 2 | 5.10% | | x |

**Table S1.** *Cont.*

| Protein Name [1] | Uniprot Accession | Name | Mass kDa | NSAF [2] | Number Unique Peptides | % Coverage | BNYVV-IV (C2) [3] | BNYVV-IV (C3) [4] |
|---|---|---|---|---|---|---|---|---|
| **Unknown or other** | | | | | | | | |
| **RS2 protein** | **Q9FNT2** | **Rs2** | **16** | **1.40E−04** | **7** | **33%** | **x** | **x** |
| Putative uncharacterized protein | E2DN06 | | 15 | 1.20E−04 | 2 | 16% | x | x |
| Histone H3.3C | P02301 | H3f3c | 15 | 9.80E−05 | 5 | 51% | x | x |
| Putative uncharacterized protein | E2DN04 | | 15 | 3.40E−05 | 2 | 13% | x | |
| 37 kDa inner envelope membrane protein, chloroplastic | P23525 | | 39 | 1.70E−05 | 3 | 14% | | x |
| Uncharacterized protein | K4PYU2 | | 18 | 8.00E−06 | 2 | 13% | | x |

Footnotes: [1] Proteins are organized based on GO terms and putative annotated function. [2] NSAF: Normalized spectral abundance factors. [3] C2: Proteins that were identified from the susceptible sugar beet genotype (R30_rz1) infected with the resistance breaking BNYVV strain from the Rockwood 158 field, Imperial County, CA (BNYVV-IV). [4] C3: Proteins that were identified from the susceptible sugar beet genotype (R30_rz1) infected with A-type of BNYVV (BNYVV-A) strain. Proteins in **bold** lettering were previously identified in the study by Larson *et al.* [1].

## References

1. Larson, R.L.; Hill, A.L.; Nunez, A. Characterization of protein changes associated with sugar beet (*Beta vulgaris*) resistance and susceptibility to *Fusarium oxysporum*. *J. Agric. Food Chem.* **2007**, *55*, 7905–7915.

© 2014 by the authors; licensee MDPI, Basel, Switzerland. This article is an open access article distributed under the terms and conditions of the Creative Commons Attribution license (http://creativecommons.org/licenses/by/3.0/).
